# No evidence of fluctuations in daily step count between infusions in people with multiple sclerosis treated with anti-CD20 monoclonal antibodies

**DOI:** 10.1177/20552173251329817

**Published:** 2025-03-25

**Authors:** Valerie J Block, Kyra Henderson, Shane Poole, Gabby B Joseph, Jeffrey M Gelfand, Bruce AC Cree, Riley Bove

**Affiliations:** Department of Neurology, Weill Institute for Neurosciences, 8785 University of California San Francisco, San Francisco, CA, USA; Department of Physical Therapy and Rehabilitation Science, University of California, San Francisco, San Francisco, CA, USA; Department of Neurology, Weill Institute for Neurosciences, 8785 University of California San Francisco, San Francisco, CA, USA; Department of Neurology, Weill Institute for Neurosciences, 8785 University of California San Francisco, San Francisco, CA, USA; Department of Radiology and Biomedical Imaging, University of California, San Francisco, San Francisco, CA, USA; Department of Neurology, Weill Institute for Neurosciences, 8785 University of California San Francisco, San Francisco, CA, USA; Department of Neurology, Weill Institute for Neurosciences, 8785 University of California San Francisco, San Francisco, CA, USA; Department of Neurology, Weill Institute for Neurosciences, 8785 University of California San Francisco, San Francisco, CA, USA

**Keywords:** Multiple sclerosis, anti-CD20 monoclonal antibodies, physical activity, step count, self-reported wearing off, activity monitoring

## Abstract

**Background:**

Patients with multiple sclerosis (MS) on some disease-modifying therapies (i.e., natalizumab), report a “wearing-off” effect characterized by increased symptoms directly before infusions. Prior research suggests this may reflect natural MS fluctuations rather than true treatment waning; however, this has not been confirmed for anti-CD20 agents (e.g., ocrelizumab). Daily step count (STEPS) can reflect overall function. This study examined temporal associations between anti-CD20 therapy infusions and STEPS.

**Methods:**

Retrospective analysis evaluated data from two Fitbit-monitored cohorts (*N* = 145 total, 32 anti-CD20-treated participants) across 60 treatment cycles. Monthly STEPS were recorded directly pre- and three-month post-infusion over the six-month treatment intervals. Mixed-effects models evaluated the relationship between infusion timing, STEPS, and participant demographics, controlling for confounding variables.

**Results:**

No significant difference in STEPS was observed pre- versus post-infusion (*p* = 0.32). An average decrease of 3.3% was noted post-infusion but was not statistically significant. No associations between STEPS and participant characteristics (e.g., age, disability level) were identified. Individual variability existed, but no clear group-level trends emerged.

**Conclusions:**

This study found no evidence of an association between timing of anti-CD20 infusion and changes in STEPS. Findings highlight the need for integrating objective measures with patient-reported outcomes and biomarkers in future research to better understand potential treatment fluctuations.

## Summary

Patients on some multiple sclerosis (MS) disease-modifying therapies (i.e., natalizumab) report a “self-reported wearing off” effect with increased symptoms before their infusion. This phenomenon lacks confirmation for anti-CD20 agents such as ocrelizumab and rituximab, via measurable MS worsening or patient-reported outcomes. A retrospective study analyzed Fitbit-monitored step counts (STEPS) in MS patients over 60 anti-CD20 therapy cycles. No significant differences in STEPS pre-, the month directly prior infusions and post-, the third month post infusion, were found. Future research integrating STEPS, biomarkers, and prospective symptom reports may clarify the “wearing-off” phenomenon, aiding understanding of its immunological basis and impact on patients.

## Introduction

Patients on some multiple sclerosis (MS) disease-modifying therapies, especially natalizumab, report a “self-reported wearing off” effect characterized by increased symptoms (primarily fatigue) in the week before monthly infusions. However, stable magnetic resonance imaging and Expanded Disability Status Scale scores suggest no associated change in detectable MS activity.^1,2^ The increased use of twice-yearly B-cell depleting therapies (anti-CD20 agents; i.e., ocrelizumab, ublituximab, rituximab) for MS over the past decade is linked to a symptomatic “self-reported wearing off” effect, with up to 55% of patients reporting a recurrence or increase in symptoms 2–4 weeks before scheduled infusion.^3–5^ Identifying an objective correlate of these reports is challenging; studies suggest that this is likely due to natural MS symptom fluctuations and interpretive bias, rather than an actual decline in drug efficacy or breakthrough activity; when using longitudinal patient-reported outcome measures, clear symptom worsening prior to reinfusion was not observed.^3,5^

Prospectively collected average daily step count (STEPS) encompasses walking capacity and holistic domains of function, such as fatigue and general well-being.^6,7^ This study investigated whether a temporal association exists between anti-CD20 therapy infusion and changes in STEPS (passively monitored via Fitbit), from two published cohort studies.^8,9^

## Methods

A retrospective analysis was conducted of people in the FITriMS study (Fitbit remote monitoring in MS, *N* = 95) and the FITMRI substudy (*N* = 50), who had completed ≥1 full cycle of anti-CD20 monoclonal antibody therapy (including rituximab or ocrelizumab). FITriMS was a prospective year-long study (enrollment: 2015–2016) monitoring physical activity, via Fitbit STEPS, in adults with MS (>18 years). Participants were able to walk for 2 min, had no documented relapses the month before enrollment, and were free from cardiovascular or musculoskeletal comorbidities impacting physical activity.^8,9^ FITMRI (2018–2019) recruited with the same criteria, monitoring STEPS over two years and including magnetic resonance imaging. All participants provided informed consent, and the University of California San Francisco institutional review board approved the protocols.

Start and end dates of Fitbit data (STEPS) were extracted, and participant electronic health records reviewed, to identify the dates of any anti-CD20 treatments completed. Participants treated with other DMTs were excluded. The first-ever cycle of anti-CD20 therapy was also excluded, and only the second and subsequent cycles were retained for analysis.

Fitbit activity data were cleaned using criteria defined in previous publications; valid days and weeks were used to calculate monthly averages.^8,9^ The weighted averages were used as a measure of monthly STEPS.

The resulting participants treated with anti-CD20 therapy during the activity monitoring epoch, the average number of infusions per person, and over the entire cohorts were recorded. To visualize activity throughout the treatment cycles, monthly STEPS were individually plotted over infusion cycles.

Daily step count for the month directly prior to the infusion (pre) was compared with the third month after the infusion (post3 M, i.e., mid six-month cycle). The second, fourth, and fifth months after infusion were also analyzed.

A linear mixed model was performed, using the Kenward–Roger Denominator Degrees of Freedom, and assuming normal distribution. An interaction term for timepoint of infusion was added to the main model to assess whether step count changes varied significantly across cycles. Finally, a series of mixed-effects models were performed to evaluate whether a change in STEPS pre and post3 M varied based on factors such as age, sex, disease duration, disability level at study entry, and MS type.

## Results

Overall, 60 valid B cell-depleting medication treatment cycles were identified for 32 adults with MS. Table 1 summarizes participant demographics (Table S1 cohort-level demographics).

**Table 1.** Demographic characteristics of participants participating in the combined studies who contributed Fitbit step count data for a total of 60 cycles while undergoing treatment with anti-CD20 therapies.

Cohort name	Combined anti-CD20 subsets
Sample size: overall N *N* (%) OCR	32 29 (90.6%)
Age at baseline, years: mean (SD)	48.9 (11.9)
Sex, Female: *N* (%)	18 (56.3%)
Disease type, progressive: *N* (%)	14 (43.8%)
Disease duration, years: mean (SD)	15.0 (11.9)
EDSS at baseline: median (range)	4.0 (1.5–6.5)
EDSS at 1 year: median (range)	4.0 (0.0–6.5)
Valid infusion cycles (*excluding first doses*) (*N*)	*60*

EDSS: Expanded Disability Status Scale; *N*: sample size; OCR: Ocrelizumab; SD: standard deviation; %: percentage of total.

* Rituximab was the only other therapy used for participants in these cohorts.

**Figure 1.**
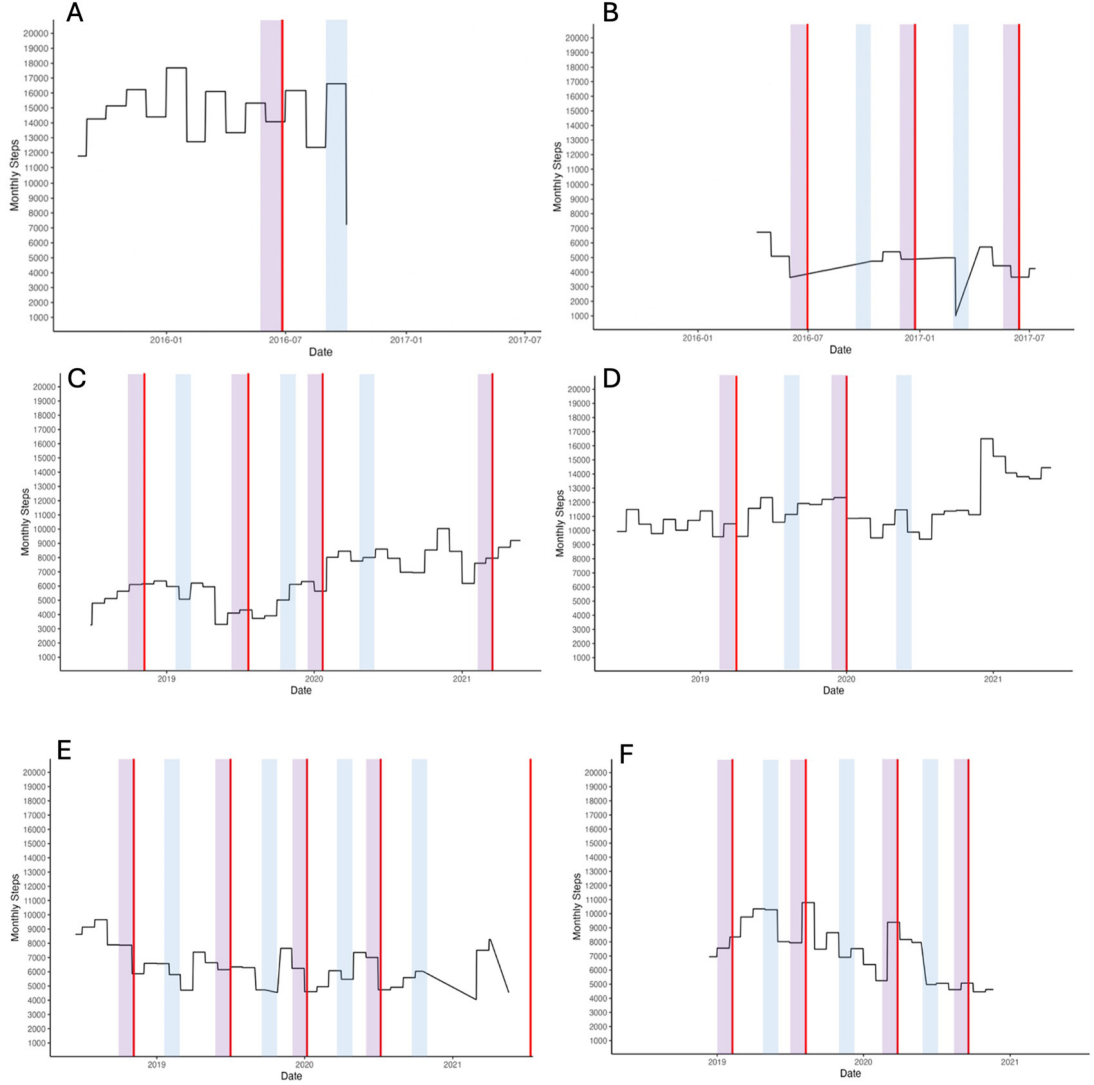
Examples of monthly step count plotted over infusion cycles per individual participant. Each graph, A–F, represents an individual participants average monthly step count over the duration of the study. The red vertical line represents the date of the anti-CD20 mAb infusion. Highlighted in magenta is the month prior to the infusion, and highlighted in light blue is the third month after the infusion date.

Visual plots of STEPS over infusion cycles (Figure 1) did not reveal clear evidence of a difference in STEPS before anti-CD20 therapy infusions. Variability existed: in some cases (Figure 1A), STEPS decreased pre-infusion (red line), however, the change was small (<1800 steps) relative to an average of ∼14,800 steps/day. Other cases (Figure 1F) showed higher pre-infusion STEPS for the first infusion, reversing in subsequent cycles.

Statistical analysis found no significant difference in STEPS between pre- and post3 M (*p* > 0.05), with confidence intervals encompassing zero (Table S2). Linear mixed model confirmed no significant difference (Coefficient = −185.11; STEPS 95% CI = −556.56, 186.24; *p* = 0.32). Interaction analysis in a mixed-effects model and visual assessments showed no significant variation in step count changes across cycles (*p* = 0.31), with no consistent trends of improvement or decline in individual patient data. When evaluating percent difference in STEPS, there was an average relative decrease of −3.3% (95% CI: −9.73%, 3.11%) after infusion compared to the pre-infusion timepoint. No significant interaction was observed between timepoint and age, sex, disease duration, MS subtype, or disability (all *p* > 0.05).

## Discussion

This study, utilizing longitudinally collected ambulation data spanning 60 infusion treatment cycles, observed no significant changes in STEPS relative to the timing of anti-CD20 infusions.

A slight decrease in STEPS was observed at the post3 M timepoint, consistent with previous cohort-level data showing a decline over time. Over six months, changes in STEPS were consistent with those seen in the broader cohort.^9^ No evidence of a pronounced decrease in STEPS was associated with the timing of infusions, which would indicate a strong “wearing-off” effect.

The lack of a statistical difference in STEPS does not rule out the possibility of subtle or individual-level associations between treatment timing and function. While some participants may have experienced a wearing-off effect reflected as reduced STEPS, this was not evident at the group level (*p* = 0.32). These study results—32 participants, 60 infusions—may reflect a limited power to detect an association.

An online survey of people with MS taking monoclonal antibody therapy reported a 27.6% completion rate, with 48% citing increased fatigue as a common self-reported wearing off symptom, and 20% considering a DMT switch.^4^ Literature shows depression and elevated body mass index as factors associated with self-reported wearing off symptoms, while biological correlates remain elusive.^1,3,4^ Without clear biological markers or consistent symptom patterns, normal symptom fluctuations could be incorrectly attributed to treatment intervals given the cyclic nature of infused anti-CD20 therapy.

Limitations of this analysis include the absence of participant-reported data about symptomatic self-reported wearing off and a small sample, and a lack of data on individual efforts to exercise or modify behavior. Our patients may not have experienced symptomatic self-reported wearing off or may not be representative of individuals who do. Although patient-reported symptoms are clinically important, they measure a distinct concept from objective changes in STEPS. We believe that additional features such as minute-by-minute step count data to assess capacity versus behavior, activity bouts and intensity levels, sleep patterns, and day-to-day variability may provide further insight. Nonetheless, our findings suggest no clear temporal pattern or decline in STEPS relative to the timing of B-cell depleting therapy infusions.

Future studies should integrate objective measures, patient-reported symptoms, and other biomarkers (e.g., CD19 levels, inflammatory serum biomarkers) to explore immunological mechanisms behind “wearing-off” effects and offer insights into patients’ lived experience.

## Data Availability

Anonymized data used for this study are available from the corresponding authors on reasonable request.
